# Nasolabial Cyst: A Case Report with Ultrasonography and Magnetic Resonance Imaging Findings

**DOI:** 10.1155/2017/4687409

**Published:** 2017-06-11

**Authors:** Ali Ocak, Suayip Burak Duman, Ibrahim Sevki Bayrakdar, Binali Cakur

**Affiliations:** ^1^Department of Oral and Maxillofacial Radiology, Faculty of Dentistry, Atatürk University, Erzurum, Turkey; ^2^Department of Oral and Maxillofacial Radiology, Faculty of Dentistry, Inonu University, Malatya, Turkey; ^3^Department of Oral and Maxillofacial Radiology, Faculty of Dentistry, Osmangazi University, Eskişehir, Turkey

## Abstract

Nasolabial cysts are uncommon nonodontogenic lesions that occur in the nasal alar region. These lesions usually present with asymptomatic swelling but can cause pain if infected. In this case report, we describe the inadequacy of conventional radiography in a nasolabial cyst case, as well as the magnetic resonance imaging (MRI) and ultrasonography (US) findings in a 54-year-old female patient.

## 1. Introduction

A nasolabial cyst is a benign, slow-growing, nonodontogenic, primarily unilateral, extraosseous soft tissue lesion located in the nasal alar region below the nasolabial fold. The pathogenesis of nasolabial cysts is uncertain; however, there are two main theories. Some authors suggest that these lesions originate from displaced epithelium of the nasolacrimal duct remnants, while others suggest that it is a developmental fissural cyst originating from epithelial remnants entrapped between the lateral nasal, globular, and maxillary processes [1]. These cysts usually occur unilaterally (90%), but bilateral lesions have been reported [2, 3]. The age of detection ranges from 12 to 75 years old; however, there is a peak incidence noted in the fourth and fifth decades of life, with a female predilection of nearly 3 : 1 for these cysts [1]. Clinically, a nasolabial cyst is characterized by a painless floating mass in the nasolabial sulcus, causing upper lip elevation and a loss of the nasolabial fold [4]. Although pain is not a frequent finding, it can occur if the cyst becomes infected. Numbness and loosening can be seen in the upper incisor teeth, as well as rupture and spontaneous drainage into the nasal and oral cavities, difficulty in nasal breathing, nasal blockage, postnasal drip, or rhinorrhea [5].

Nasolabial cysts cannot be seen on conventional radiography if there are no associated bone changes. However, these cysts may be aspirated and injected with a contrast agent for better visibility on plain radiographs [3, 5, 6]. In addition, computed tomography (CT) can show a well-demarcated, rounded, homogeneous, low-density soft tissue lesion in the nasolabial region. Evidence of scalloping and bone remodeling may also be depicted [3, 6, 7]. Magnetic resonance imaging (MRI) can show the characteristics of a liquid-containing cyst, with low intensity on the T1-weighted images and high intensity on the T2-weighted images [2, 3]. Ultrasonography (US) can reveal the cystic nature of these lesions, for example, well circumscribed, rounded, or oval shapes and anechoic fluid-filled masses in the nasolabial sulcus region [8, 9].

The purpose of this study was to report the case of a nasolabial cyst and to describe its USG and MRI exam features.

## 2. Case Report

**Figure 1:**
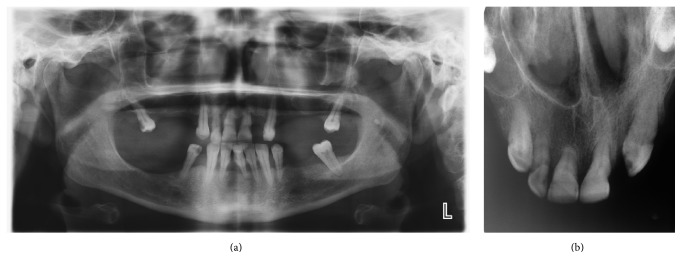
Patient's panoramic (a) and occlusal (b) radiographies show no bone changes on apical region of upper incisor teeth.

**Figure 2:**
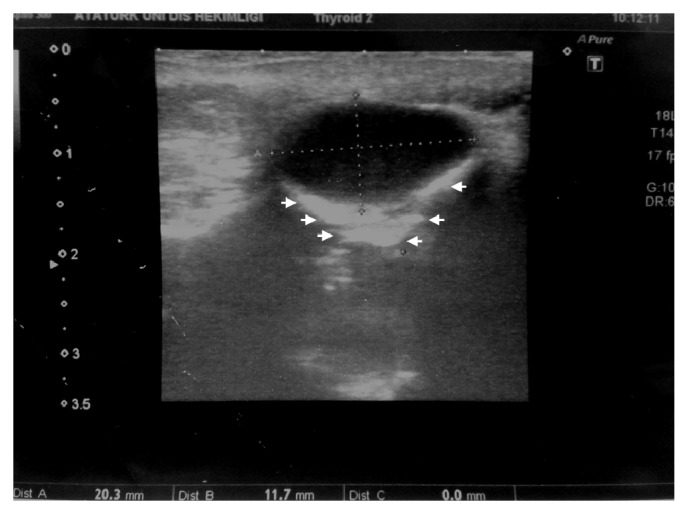
Ultrasonography shows well-defined anechoic cystic lesion about 2 cm diameter with posterior acoustic enhancement (arrows).

**Figure 3:**
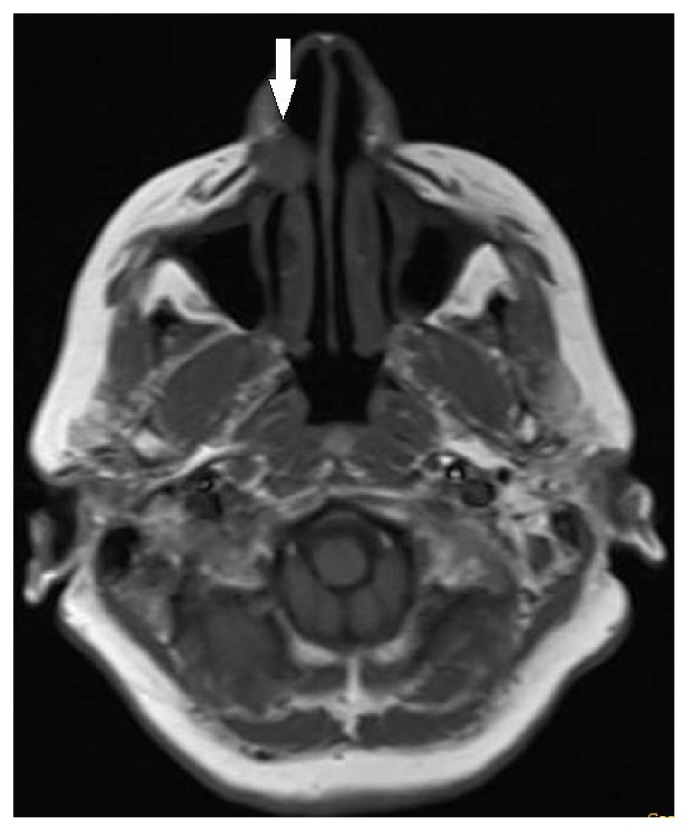
The axial T1-weighted MR image shows the lesion with a homogeneous hypointensity (arrow).

**Figure 4:**
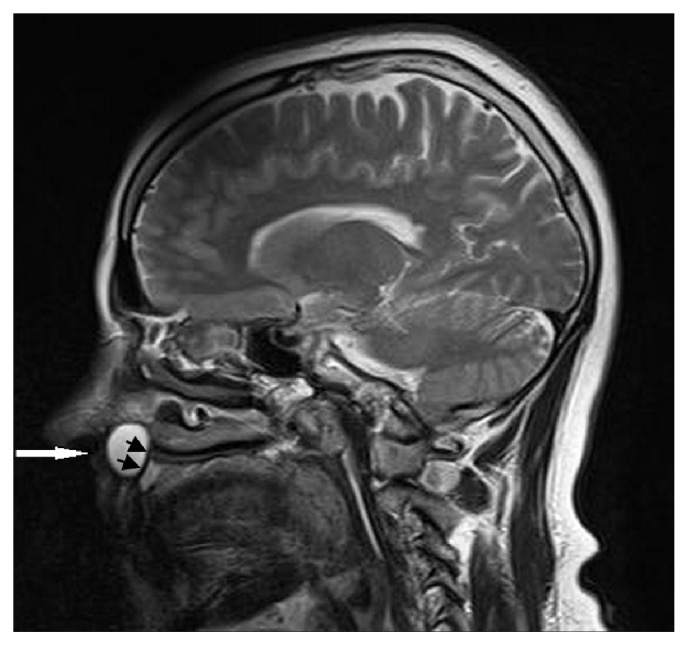
The sagittal T2-weighted MR image shows hyperintense lesion in the lower nasal fossa (white arrow) and bone erosion (black arrows).

**Figure 5:**
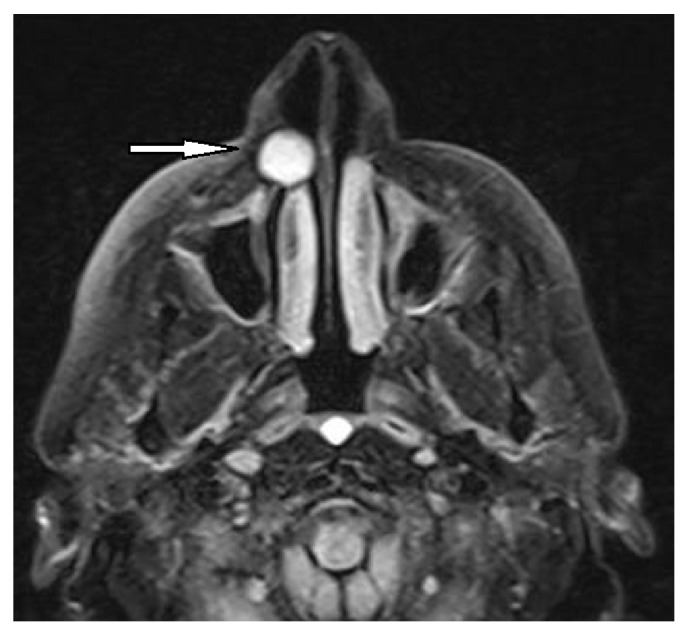
The axial T2-weighted fat-saturated image shows the well circumscribed lesion with a homogeneous hyperintensity (arrow).

A 54-year-old woman presented to our department with a common toothache. She described a history of a neurological examination with a brain MRI, and the neurologist sent her to us due to the possibility of a dentally originating lesion. During the clinical examination, we noticed a palpable fluctuant swelling on the upper labial sulcus, beneath the right nasolabial fold. There were no bony changes in the orthopantomography and occlusal radiography examinations, and all of the associated teeth were shown to be vital using electrical pulp testing (Figure 1). US was performed, and a well-defined, ovoid shaped, approximately 2-cm in diameter anechoic cystic lesion was observed (Figure 2). The MRI revealed a well-defined, round, cystic mass in the right nasal alar region. The relevant cystic lesion exhibited a homogeneous hypoisointense (with adjacent soft tissue) appearance on the T1-weighted images and a homogenous hyperintense appearance on the T2-weighted images (Figures 3, 4, and 5).

Based on the MRI, US, plain radiographic, and clinical findings, a preliminary diagnosis of a nasolabial cyst was made. The lesion was removed surgically with an intraoral approach under local anesthesia, and the surgical specimen was sent for a histopathological examination. The lesion was diagnosed as a nasolabial cyst.

## 3. Discussion

Nasolabial cysts have been known by many names. First described by Zuckerkandl in 1882, subsequent names appeared, such as Klestadt's cyst, nasal alveolar cyst, nasal wing cyst, and mucoid cyst of the nose; finally, Rao used the term “nasolabial cyst” as a more correct definition, which has remained in use until today [5]. It is important to the vitality of the adjacent teeth that an infected nasolabial cyst may simulate an acute dentoalveolar abscess [1]. A differential diagnosis should be made with those lesions that mimic the same clinical appearance when located in the upper lip, including tumors originating in the salivary gland, such as pleomorphic adenoma, canalicular adenoma, mucoepidermoid carcinoma, adenoid cystic carcinoma, and polymorphous low-grade adenocarcinoma [6].

Nasolabial cysts are rare extraosseous developmental lesions that occur beneath the upper lip and adjacent to the alveolar process, above the apices of the incisors [3]. They are usually unilateral, but bilateral lesions have been reported, at a rate of approximately 10% of the cases [2, 3]. Clinically, nasolabial cysts are asymptomatic lesions but may grow large and also extend inferiorly into the labial sulcus, with an elevation of the nasal floor and tumefaction seen in the oral cavity [10]. These cysts can range in size from 1 cm to 5 cm and can lead to the erosion of the underlying bone if they do grow to a large size [11].

For the diagnosis of these lesions, several imaging methods can be used. Although plain radiographs may not show any detectable changes if there is no bone erosion, after the aspiration of the cystic fluid, the cyst can be injected with contrast material for better visibility [3, 5, 6]. In this case, the cyst was about 2 cm in size, and there were no bone changes in the panoramic and occlusal radiographs; however, the bone tissue underwent erosion on the lesion side, as seen on the sagittal MRI sections. A CT scan can show high contrast resolution and provide good bone and soft tissue definition [12]. Moreover, CT imaging is preferable to MRI due to its lower cost, although the MRI provides excellent soft tissue contrast resolution without ionizing radiation. Kato et al. reported that the MRI scans of nasolabial cysts showed various signal intensities, especially the T1-weighted images, due to the different viscosities of the intracystic fluid [13]. In a number of studies, the MRI findings of nasolabial cysts in the T1-weighted images showed hypointensity to intermediate intensity and the T2-weighted images showed hyperintensity [4, 14, 15]. We obtained the same findings on the MRI scan that we reported here. Some studies have reported that there is no enhancement of the contents or the wall of the cyst after contrast-enhanced MRI [4, 13]. However, we did not use contrast material in our case.

US is a diagnostic method that is often used for the examination of soft tissue lesions, and it can be used successfully in the maxillofacial region. US has some advantages over MRI and CT, like its nonionized characteristics and the fact that it is inexpensive [8]. Nasolabial cysts are soft tissue lesions that can be diagnosed using US [8], which can also be used for the diagnosis of cellulitis and abscesses in the maxillofacial region [8]. In addition, it is helpful in the evaluation of the lymph node metastasis of oral cancer, vascular structures, and salivary gland diseases, as well as in injection biopsies [8]. Overall, US is a valuable modality for the differential diagnosis of cysts, tumors, and soft tissue swelling in the cervicofacial region [8]. In their case, Acar et al. reported that US showed a well-defined anechoic cystic lesion beneath the nasolabial fold [8]. We observed a similar appearance: an approximately 2-cm in diameter anechoic, ovoid, and well-defined cystic lesion.

The treatment of choice remains simple: enucleation with an intraoral sublabial approach and transnasal marsupialization, which has been performed with successful results. After careful and complete surgical treatment, recurrence is exceedingly rare [6].

## 4. Conclusion

Nasolabial cysts are soft tissue lesions; therefore, conventional radiographs are often inadequate. Additional imaging modalities and clinical examinations are needed to diagnose them correctly. US and MRI are successful diagnostic imaging methods for evaluating the location, determining the contents of the cyst, and diagnosing, alongside a clinical examination. It may be advisable to use a combination of MRI and US in the diagnosis of nonosseous soft tissue lesions, such as nasolabial cysts.
